# Income and conversion handicaps: estimating the impact of child chronic illness/disability on family income and the extra cost of child chronic illness/child disability in Ireland using a standard of living approach

**DOI:** 10.1007/s10198-021-01371-4

**Published:** 2021-09-09

**Authors:** Áine Roddy

**Affiliations:** Care Policy and Evaluation Centre, Department of Health Policy, London School of Economics & Political Science, London, UK

**Keywords:** Child disability, Family income, Household standard of living, Extra cost of disability, Economic hardship, C31, I31, J14

## Abstract

Child chronic illness/ disability can present significant challenges for children, families and society that require appropriate policy responses; yet little is known about the demands placed on families resources from an economics perspective in terms of its impact on household income and the extra income required to achieve the same standard of living as families who do not have a child with a chronic illness/disability. The paper uses data from the *Growing Up in Ireland National survey* dataset for nine year olds. It is the first study to empirically investigate the impact of child chronic illness/disability on earnings, standard of living and the extra cost of disability together. It is also the first study to explicitly address endogeneity in the standard of living model by using a two-stage process where residuals were harvested to provide efficient estimates. The findings show that families experience significant disadvantage and economic hardship due to reduced household income and a lower standard of living due to the extra cost of disability that would require considerable income to compensate. Policy implications of these findings suggest that a tiered approach to disability support payments which encompass broader criteria for inclusion based on varying severity levels be introduced to alleviate the financial hardship and compromised economic wellbeing of families affected. In addition, more innovative policies are required to implement appropriate timely access to health and social care services and flexi parental employment, which in turn requires the provision of adequate access to high quality educational and care facilities.

**Supplementary Information:**

The online version contains supplementary material available at 10.1007/s10198-021-01371-4.

## Introduction

Additional needs associated with behavioural, developmental and physical child chronic illness or disability such as the time costs of caring for a child who may be distressed or unwell and needs constant supervision and support, attending medical appointments, out-of-pocket expenditure and psychological impact can impinge upon many dimensions of family wellbeing [1, 2]. These impacts manifest into the concept of family spillover, whereby the demands placed on family resources as a result of caring for a child with a chronic illness or disability often result in adverse family outcomes [3, 6]. Nevertheless, the existing literature tends to concentrate on exploring the effect of spillover on the primary caregiver and family members health status (Ibid). Given the demands placed on trying to sustain employment and manage family responsibilities, it is important to establish what are the economic spillover effects of caring for a child with a chronic illness or disability with regard to generating a household income and maintaining a standard of living. These economic aspects are of particular relevance to this paper for two reasons. First, previous studies have identified the protective mechanisms that income can provide when health shocks occur [7]. Second, families caring for a child with a chronic illness or disability require additional resources to address disability-related needs, which are subject to household income constraints [2, 8]. Consequently, Sen [9] identifies two specific challenges in relation to individuals with disabilities that are over and above those faced by others. Taken together, these increase the risk of living in poverty. First, an earnings handicap in the form of barriers to entering and sustaining employment and; second, the conversion handicap, which arises as a result of diverting their limited income to considerable additional and on-going expenses associated with the cost of disability. Combined, these result in a lower standard of living for the family. Sen’s [9] term “coupling of disadvantages” is used to describe the “earning handicap” and “conversion handicap” faced by disabled individuals. While previous research has empirically investigated the impact of child and adult disabilities on (1) earnings and (2) the extra cost of disability using the standard of living (SoL) approach, no study has examined both in the same paper. Furthermore, there is a paucity of literature on the extra cost of child disability using the SoL approach [8, 10–12]. Only four previous studies have used this method to estimate the extra cost of childhood disability—three in the UK [8, 10, 11] and one in Vietnam [12]. It should be pointed out that only one of the studies estimated the extra costs based on the degree of severity to which the child experiences daily limitations and in this ambiguity existed regarding the measurement of severity [10].

Child disability policies in Ireland are currently shaped by the Disability Act (2005) [13]. This entitles children with a disability to have their needs assessed and to receive special health and educational publicly funded services in line with their needs. However, due to a lack of service provision, this entitlement does not necessary mean children with disabilities will receive these services. This results in significant unmet needs or parents paying privately for services and supports often leading to financial hardship and debt [14, 15]. The *Progressing Disability Services for Children and Young People Programme* (2020) [16] is underpinned by a generalized approach that focuses on needs rather than a diagnosis based approach to access services, however, this is subject to criticism as a specialized approach from the outset may be more beneficial for certain conditions, e.g. autism.

This paper addresses these gaps in knowledge using the *Growing Up in Ireland* national dataset to focus on four key research objectives (1) concentrating on isolating the impact of child chronic illness/disability on family income using an ordinary least-sqaures model, (2) addressing conversion issues resulting in a lower standard of living by examining variations in need across families with and without a child with a chronic illness/disability using a standard of living modelling approach (3) estimate the extra cost of disability in monetary terms based on the relationship between household income and variations in standard of living for families with and without a child with a chronic illness/disability, (4) explicitly address endogeneity in the standard of living model to provide efficient estimates.

The key contributions of this paper are (1) it is the first study to empirically investigate the impact of child chronic illness/disability on earnings, standard of living and the extra cost of disability together, (2) it is the first study to explicitly address endogeneity in the SoL model using a two-stage process where residuals were harvested to provide efficient estimates (3) provides evidence for policymakers regarding the increased likelihood for economic hardship and the need to design appropriate policies and interventions based on the degree to which the child is hampered on a daily basis in light of the economic impact of child chronic illness/disability on family income, standard of living and the extra cost of child chronic illness for each category.

## Conceptual framework

The conceptual framework underpinning this empirical work is the standard of living approach theory for disability that was first implemented by Berthoud et al*.* [17]. The SoL approach for disability is distinguished within the literature as a measure of material wellbeing as opposed to utility [18]. Essentially, it provides equivalization^1^ of household income which takes into account household structure and size, in addition to factors which influence variations in household needs, e.g. disability. The SoL approach seeks to quantify the cost of meeting these needs by looking at how much money the household would be required to be given to bring it to a comparable standard of living as a household that is not obliged to meet these needs, *ceteris paribus*. The SoL approach has previously been used to measure the standard of living of groups with higher needs [19]. Berthoud et al*.* [17] argue that because of household income constraints, the true ‘cost of disability’ is in fact reflected in the reduced expenditure on other goods and services as a result of families that have a household member with a disability diverting their scarce income to pay for disability-related needs.

In a broad sense, these needs can be defined as the capacity to benefit from a health/social care intervention; a desire and ability to benefit from disability-related goods and services, i.e. assistive technology, special diet, private therapies, etc. Berthoud [20] raises an interesting point in relation to defining ‘need’. He identifies both ‘horizontal equity’ and ‘vertical equity’ issues in the context of goods and services being identified with the former, while lack of income is associated with the later. Disability-related needs for goods and services are relatively income inelastic, those with limited incomes tend to prioritize a high proportion of their income on these needs, whilst having to forfeit their general standard of living through reduced consumption of other goods and services due to their budget constraints. Whilst it is considered reasonable to assume that expenditure patterns on disability-related needs will increase and not remain fixed when income increases, the authors consider that “the true cost is the one actually experienced by this person with this income” [20]. A limitation of this methodology is that it does not address the issue of unmet disability needs relating to not being able to consume a sufficient quantity of disability-related goods and services or the opportunity costs associated with informal care [21].^2^ Furthermore, it does not address the issue of intrahousehold allocation (child’s needs may be met, but not the parents basic needs) or personal wellbeing. This suggests that two households may report the same standard of living, but they may not have the same allocation of resources to each type of household member. While the needs of parents with and without a child with a disability may differ in some regards, there is a susceptibility that parents of children with disabilities may lower their own expectations in their standard of living to compensate to meet the needs of their child with a chronic illness/disability.

The theoretical underpinnings of the approach set out to measure the reduction in the general standard of living of families that have a household member with a disability, as distinct from the total extra cost of disability. This requires the usage of a suitable indicator of the families standard of living which must be “positively related to income and is not directly influenced by disability, but which responds to the extra costs of disability in the manner just outlined” [17] (1993, p. 83). Further details on the chosen indicator are outlined in the methods sections of this paper. As expected, an increase in income for both non-disabled and disabled households is associated with a higher standard of living. Following Zaidi and Burchardt [21], the cost of disability is represented algebraically as follows:

1 $$ S = \alpha Y + \beta D + yX + k, $$

where *S* represents the SoL indicator, *Y* is household income, *D* is the disability status of the study child, *X* is a vector of other characteristics, and *k* is the intercept term which represents a minimum level of standard of living. Thus, the extra cost of disability, denoted as *E*, is calculated such that:

2 $$E\, = \,dY/dD\, = - \beta /\alpha$$

$\beta$ represents the distance between the income for households that have a child with a disability and households without a child with a disability. $\alpha$ is used to estimate the slope.

## Data and methods

### Data

The Growing Up in Ireland (GUI) national study is a two-stage clustered survey of 8568 children aged between 8 and 10 years of age,^3^ their caregivers, teachers and school principals in the Republic of Ireland. The analysis is based on Wave 1 for 9 year olds (2007–2008), where the primary sampling unit was 910 schools. The population frame used was based on a list of all mainstream, special and private schools in Ireland provided by the Department of Education and Science (3246 schools). All Irish children have a constitutional right to free education. Irish policy on educational placements for children with disabilities falls under the Education for Persons with Special Educational Needs (EPSEN) Act 2004 [23]. The EPSEN Act acknowledges that special educational needs may present as a result of physical, sensory, mental health and learning disability. Inclusive educational environments where children with a disability are educated with children without special educational needs is considered central to the EPSEN Act, with two exceptions—unless it is not in the best interest of the child or is not in the best interest of the other children in the class. Parents have a choice subject to the exceptions whether they want to send their child to a mainstream class, special class or a special school in Ireland. However, significant challenges are being experienced by parents finding appropriate schools for their children, resulting in some children with disabilities being put on short days (sent home early from school) [24, 25]. The GUI dataset was chosen as it is the only Irish dataset^4^ that has sufficient information about a child’s chronic illness/disability and the other required socioeconomic variables necessary for the analysis. At the time of analysis, the 2007–2008 GUI dataset was the most recent dataset available for this age category in Ireland. It is important to note a number of previous studies which applied the standard of living approach used datasets of a similar age. For example, Cullinan et al. (2013) [26] used data from the Living In Ireland Survey 2001; Morciano et al. [27] used data from the UK family Resources Survey 2007/08; Saunders (2007) [28] used the Household Expenditure Survey 1998–1999.

It was deemed appropriate to use cross-sectional data rather than longitudinal data to avoid capturing the impact of the economic recession on households’ standard of living in Wave 2. An analysis of trends in economic stress and the great recession in Ireland based on the Central Statistics Office (CSO) Survey of Income and Living Conditions (SILC) data, reported the Irish populations’ difficulty in making ends meet rose from 21.3% in 2007 (GUI Wave 1 data collection) to 32.2% in 2011 (GUI Wave 2 data collection) [29]. Given that the difficulty in making ends meet variable was used as the standard of living indicator in this study, the inclusion of the second wave of data would likely have disproportionately captured the impact of the economic recession on participants more than the impact of the chronic illness/disability. Furthermore, there was a considerable reduction in the number of children with a chronic illness/disability from Wave 1 (863 children) to Wave 2 (765 children), which may impact upon both the sample size and composition—attrition being unlikely to be purely random as the time involved in participating in the study may have been too much of a burden for families that have a child with a chronic illness/disability. In addition, most of the previous studies which applied the standard of living approach implemented a cross-sectional approach, thus for comparative purposes, a cross-sectional approach was more relevant. While there are limitations regarding the usage of cross-sectional data in terms of not being able to make causal inferences or measure the impact as the children age, in the context of this study and for the reasons outlined above, cross-sectional data were deemed suitable to address the research objectives.

Household and School-based survey instruments were used to obtain the data using CAPI software (main sections) and self-completion paper based questionnaire (sensitive sections) in the presence of trained interviewers from the ESRI. The dataset includes approximately 14% of all 9 year olds in the Republic of Ireland in 2008. See [30] for an in-depth guide to the datasets and survey methodology. The study received ethical approval from the Health Research Board’s Research Ethics Committee based in Dublin, Ireland which was performed in accordance with the ethical standards laid down in the 1964 Declaration of Helsinki and its later amendments. The study child and parent/guardian provided written informed assent and consent prior to their inclusion in the study.

### Description of variables—dependent variables

The following dependent variables are used in two separate models which estimate the income penalty and estimate what the additional income is to compensate for the lower standard of living incurred by families with a child with a disability—(1) Weekly household income (linear) (2) Difficulty making ends meet.

### Weekly household income

The weekly household income^5^ model will test the hypothesized income penalty to see to what extent the presence of child disability is adversely associated with household income. Given the trade-offs faced by households when deciding how to allocate time [32], households caring for a child with a chronic illness/disability often face additional increased non-market labour responsibilities as a result of having to devote time and energy to their child’s special needs, resulting in reduced labour supply. Income rather than equivalised income was used in the income analysis. Given that households containing a child with a disability will differ in terms of demands on household resources to those of households with non-disabled children, the use of “standard” equivalisation weights to adjust for household size and composition would directly bias the income measure used and thought not to be appropriate. Instead size and household structure was controlled through the use of covariates.

### Standard of living indicator—difficulty making ends meet

The second dependent variable representing households having difficulty making ends meet was used as an indicator of standard of living to implement the SoL approach [17]. The difficulty in making ends meet dependent variable was constructed based on the following statement and closed ended question being posed to the primary caregiver in the household.

*A household may have different sources of income and more than one household member may contribute to it. Concerning your household’s total monthly or weekly income, with which degree of ease or difficulty is the household able to make ends meet?*

Answer options (tick one only) with *great difficulty/with difficulty/ with some difficulty/ fairly easily/ easily/ very easily*”.^6^ [33]

Previous studies have used a range of SoL indicators. Following previous studies [21, 28, 34, 35] this subjective indictor was chosen as it was considered most suitable to capture the current financial situation of the household. Furthermore, it is a strong indicator of the households’ standard of living. A limitation of the study is that the dataset does not contain another suitable SoL indicator for robustness purposes.

### Explanatory variables

#### Definition and measurement of child chronic illness/disability

The primary objective of this paper is to isolate the impact of childhood chronic illness/disability^7^ on the two economic wellbeing dimensions considered. Given that there is no definitive definition of child disability in Ireland [36], this paper defines childhood chronic illness/disability as measured within the GUI dataset based on a parental response (primary caregiver) to the interviewer’s following question – *Does the Study Child have any on-going chronic physical or mental health problem, illness or disability?*^8^

1. Yes
2. No

If Yes, this is a binary variable with the value 1 if the child has a chronic illness/disability and 0 otherwise. Furthermore, the primary carer is asked by the interviewer to rank the degree to which the child is hampered by their chronic illness/disability on a daily basis as follows:

Is the Study Child hampered in his/her daily activities by this problem, illness or disability?

1. Yes, severely
2. Yes, to some extent
3. No

These are all separate binary variables with 1 representing the appropriate response while the comparison group is based on not having a child with a chronic illness/disability. Details on the type of conditions the children have are listed in Tables S3-S5 in accordance with ICD-10 criteria.

Three categories of covariates which were pertinent to the hypothesized “income handicap” model were included—child, parental and household level characteristics. Each of these variables influence the households ability to generate resources. A description of all the variables included in the model are provide in Table S8.

The explanatory covariates tested in the second econometric model based on the SoL indicator dependent variable ‘Difficulty making ends meet’ addresses “the conversion handicap”. To understand the exact relationship between standard of living and household income it was necessary to test which income specification variable best fitted the model.^9^ The weekly linear income variable and income squared variables were based on the residuals of the OLS model in order to perform two-stage residual inclusion estimation to address endogeneity in the SoL model. Neither of the income variables were endogenous for the reasons detailed above. The rationale for the inclusion of the other explanatory covariates which are based on child, parental and household level characteristics is to control for other sources of variation in household needs that may impact upon standard of living. Details of these variables are presented in Table S9.

### Descriptive statistics

The characteristics of the households in the GUI dataset who have a study child with and without a chronic illness/disability are compared. Sampling weights are applied to estimate the descriptive statistics to make the sample representative of the structure of the target population—the Irish population [37]. The weighted and unweighted characteristics of the study sample are presented in Table S6 and Table S7 in the supplemental section. The choice of social, economic and demographic variables chosen were due to the influence they had on a household’s ability to generate resources and to examine variations in households needs that may impact upon SoL. Parental ethnicity is included as language barriers and racism may hinder employment opportunities and acts as a proxy for possible differences in expectations between the indigenous population and migrants of what is a reasonable SoL. Maternal depression was included, because mothers tends to be the primary carer for the child with a chronic illness/disability and it reflects caregiver strain associated with raising a child with a disability and may influence the subjectiveness of their SoL response. Equivalised income was included to account for the number of people in the household.

### Econometric model specifications

The multivariate analyses presented in this paper are based on a unified modelling approach to conduct two-stage residual inclusion estimations to address potential endogeneity using the following econometric models—Ordinary Least Square (OLS) models and probit models [38]. The analysis is cross-sectional in nature, therefore, it is important to set out that causal inferences cannot be drawn from the findings. Weighted and unweighted OLS and probit models were estimated [37], to address potential sources of bias related to use of unweighted data. A boxcox test was performed on the OLS model to determine the most suitable functional form for the income dependent variable (linear, log, and multiplicative inverse). This approach addresses potential skewness by transforming the dependent variable to log format, while maintaining the normal assumptions requirement [39]. The null hypothesis results rejected each of the three potential models, thus the general theta coefficient was interpreted to support a linear model. Cluster-robust standard errors were applied to account for the design effect of the sampling strategy. The school ID variable was used to control for correlations in model errors within-cluster groups to avoid overstating estimates [40]. For instance, all the children in the sample were recruited at a school level; therefore, children with a chronic illness/disability attending the same school may be influenced by unobserved factors such as timely access to healthcare in that area, environmental factors such as pollution, disadvantaged areas, etc. which may impact upon their health status and result in within-cluster error correlations.

### Two-stage process of residual harvesting

To address potential endogeneity in the SoL model arising from the income variable, a two-stage process where residuals are harvested from the income equation (OLS model) and used to integrate in the SoL equation (probit model) was implemented [38]. In essence, the approach suggests that some part of the variation in income is explained by disability (for example, transfer payments or forgone employment opportunities) and part is not explained (“usual” income by the principal wage earner)—the residual. That part which is not related to disability is used to explain income related variations in SoL, reflecting, for example, the principle or usual resources available to the household. Central to this approach is recognising that there are potentially unobserved factors at play between the income independent variables in the SoL model that are correlated with both SoL and income, resulting in endogeneity in the model. These are addressed using residuals from the linear auxiliary equation (OLS model (3)).

### The first stage

#### Linear regression model estimated using ordinary least square models

The OLS model can be specified as follows:

3 $$Y= \widehat{{\alpha }_{0}}+ \widehat{{\alpha }_{1}}D+\dots \dots \dots .{\varepsilon }_{i}$$

$$Y=\widehat{Y}+\varepsilon$$

$$Y-\widehat{Y}= \varepsilon .$$

The function captures the relationship between income and having a disabled child in the household. This could be positive due to transfer payments or negative due to lost employment opportunities. ε is income not related to child disability. For ease of exposition, as noted, the latter can be thought of as income unaffected by the vagaries of the child’s disability. Therefore, the paper estimates (3) and saves ε the residual term which is then used in the SoL equation in (4). In the OLS model, the presence of heteroscedasticity was confirmed by both visual graphs and formally by a Breusch–Pagan test where the iid assumption was not relaxed (*p* = 0.0383), thus rejecting the null. The application of cluster-robust  standard errors addressed this issue. The Variation Inflation Factor (VIF) test showed that there was only multicollinearity in the age and age squared variables in addition to the education variables for parental secondary and third level education, which did not require further action.

### Stage 2

#### Standard of living modelling approach using a probit model

Probit regression analysis were employed for the SoL model which follows that of previous literature which used a binary SoL indicator [21]. As highlighted earlier in the conceptual framework, the coefficient estimates presented in Table 3 are used to calculate the extra cost of disability by dividing the disability coefficient by the slope. Given the best fit of the model includes an income squared coefficient, the slope of the quadratic equation plugged the mean income from each income quintile for each disability category to calculate estimates. Average marginal effects are also calculated and presented for ease of interpretation of the models.

4 $$SoL= \widehat{{\beta }_{0}}+ \widehat{{\beta }_{1}}\varepsilon +\dots ..{\mu }_{i}.$$

SoL is standard of living, the observed counterpart of a latent variable ‘C’ that can be thought of as capturing how ‘comfortable’ the family are. This paper estimates (3) to ascertain Sen’s earnings handicap and (4) to estimate the conversion handicap. To check that the model was correctly specified, careful consideration was given to choosing an appropriate standard of living indicator variable. The dependent variable which represents the standard of living indicator is based on household difficulty experienced in making ends meet. Berthoud et al. (1993, p. 83) [17] note that “all this depends on our finding an indicator of people’s standard of living which is positively related to income and is not directly influenced by disability, but which responds to the extra costs of disability”. Therefore, the SoL indicator was tested empirically by regressing it on the income variable to see whether it was statistically significant and that the findings were in keeping with economic intuitive; both criteria were met. Those who had difficulty making ends meet had a -23,179.28 reduction in income, with these results statistically significant (p = 0.000).

A number of studies have chosen the exact same SoL indicator [21, 28, 34]; however, they utilised ordered logistic models because of the ordinal nature of the variable. Whilst this was the preferred approach for consistency reasons in this study, it was not possible, because omodel and Brant tests showed that the parallel regression assumption was violated by the income variables and maternal disability variables. Given the assumptions of the ordered logit model were violated, consideration was given to other models based on an ordered dependent variable. Generalized ordered logit models are considered a superior alternative to correct for the violation of the proportional odds assumption.^10^ The generalised ordered logit model approach relaxes the proportional odds assumptions by providing coefficient estimates for each of the ordered outcomes, i.e. for the income coefficient which violated the proportional odds assumption, it produces coefficients for each of the six level ‘making ends meet’ dependent variable. However, this approach is also noted for being problematic [41, 42] due to the complexity of having a number of coefficients for the explanatory variables that have violated the assumptions. In the context of this paper, it is problematic, because the income variables which are used to calculate the extra cost of disability outlined below, violated the proportional odds assumption which would make the interpretation of these coefficients estimated by generalized ordered logit models more complex and less useful for policymakers [41]. Second, the issue of potential endogeneity was addressed by implementing a two-stage process of residual harvesting [38]. In this context, it was not considered viable to proceed with the ordered dependent variable in its current format. Thus, the decision was made to use a probit model as it was the most parsimonious model, and to create a binary variable for the SoL indicator as this approach is accepted within the literature. For instance, a SoL indicator based on a binary variable for whether a household had savings or not was used in a previous study [21].

In terms of specifying the most parsimonious probit model, sensitivity analysis accounted for various income specification variables which are presented in Tables S10 and S11 in the supplemental section. Models 3 from Tables S10 and S11 were considered the most suitable based on their AIC, BIC and log-likelihood tests. Furthermore, the linktest results implied that the explanatory variables are specified correctly in that model. Tables S10 and S11 shows that the predicted squared estimates for Models 2 and 4 were statistically significant, meaning these models were not correctly specified. As previously highlighted, the actual model advances on the simple linear model set out in the conceptual framework by taking into consideration the shape of the relationship between household income, standard of living and disability. All analyses were conducted using STATA SE/14 [43]. As an additional sensitivity analysis, an alternate modelling approach was employed to address endogeneity [44]. In this income is included in addition to the residuals from the first equation as covariates in the SoL function. As alternative strategies may yield different results this was thought to be prudent. These results are presented in Table S14.

## Results

### Prevalence and severity of chronic illnesses/disability

The prevalence of chronic illness/disability within the dataset is presented in Table S1. A total of 863 children (10% of the sample) were reported by their primary caregiver as having an on-going chronic physical or mental health problem, illness or disability. Table S2 provides a breakdown of the extent to which the children are hampered on a daily basis in his/her activities by their chronic illness/disability. The availability and inclusion of variables which take account of the degree to which the child is hampered on a daily basis strengthens the analysis, nevertheless, the results for children severely hampered on a daily basis should be interpreted with caution due to the small sample size of 47. Tables S3–S5 provide information on the nature of the chronic illnesses in accordance with international classification of diseases 10 criteria.

### Results of the characteristics of the study sample

Descriptive statistics of the weighted and unweighted characteristics of the study sample are provided in Table S6 and Table S7 in the supplemental section. The results compare the characteristics of two groups—households who do not have a study child with a chronic illness/disability (*N* = 7,691) versus households who have a study child with a chronic illness/disability (*N* = 863). To test whether there is a statistically significant relationship between the categorical variables, the final column presents the Pearson chi-square test which is corrected to account for the inclusion of weights, resulting in it being converted to a design-based F statistic [46]. Weights were applied to make the sample results representative of the population the sample was drawn from. Overall, these results illustrate a consistent pattern of socioeconomic disadvantage amongst households who have a child with a chronic illness/disability, in contrast to other households.

### Results of unified modelling approach

#### Household income OLS models

Table 1 below presents non-weighted ordinary least square (OLS) estimates of the association between child disability variables and a range of independent socioeconomic variables regarding the dependent variable weekly household income. Columns I shows that households raising a child with a chronic illness/disability, ceteris paribus, experience a €96.35 level reduction in weekly household income (OLS = -96.351***). Column II compares estimates based on the degree to which the child is hampered in their daily activities by their condition. Each of the three categories of severity saw further reductions in household income, in comparison to households who did not have a child with any chronic illness/disability. For example, households that had a child severely hampered on a daily basis experienced a €260.12 reduction (though not statistically significant), households with a child who has some limitation incurred a €116.67 reduction in weekly income (OLS = -116.683***); while households with a child who has no limitation in daily activities had a €78.11 reduction in weekly income (OLS = − 78.110**). The results for the remainder of the socioeconomic variables in the models are consistent with economic intuition. The weighted OLS estimates which are very similar in findings to the non-weighted OLS results in Table 1 are presented in Table S12. As explained in the methods section, residuals were harvested from this OLS income model and used in the probit regression for the standard of living indicator model to integrate the results in a unified modelling approach. The purpose of which was to address endogeneity in the SoL model and use the coefficients to calculate the extra cost of disability, the results of which are presented below.

**Table 1:** OLS estimates for the weekly household income (non-weighted)

Variables	Model 1 OLS (I)	Model 2 OLS (III)
Children’s Health Status		
Children have no chronic illness/disability		
Children have a chronic illness/disability	− 96.351*** (26.585)	
Children have a severe limitation in daily activities		− 260.131 (170.633)
Children have some limitation in daily activities		− 116.683*** (40.299)
Children have no limitation in daily activities as a result of their condition		− 78.110** (33.003)
Mothers’ Characteristics		
* Mothers’ Age*	− 292.160 (181.544)	− 291.152 (181.910)
Mothers’ age squared	7.791* ( 4.690)	7.765* (4.699)
Mothers’ age cubed	− 0.065 (0.040)	− 0.065 (0.040)
*Mothers’ ethnicity—Irish*		
Mothers’ ethnicity non-Irish	− 43.473 (47.030)	− 43.667 (47.050)
*Mothers’ highest educational attainment*		
Mothers who have a primary education or none		
Mothers who have a secondary level education	87.114* (52.576)	86.976* (52.679)
Mothers who have a third level education or higher	292.718*** (56.574)	292.097*** (56.660)
*Mothers’ health status*		
Mothers who have a chronic illness or disability	− 38.559 (29.171)	− 39.745 (29.194)
*Mothers who are not depressed*		
Mothers who are depressed	10.320 (24.159)	10.240 (24.168)
Mothers’ not in paid work		
Mothers in part-time paid work	32.937 (66.922)	31.504 (67.163)
Mothers in full-time paid work	36.629 (26.441)	36.247 (26.484)
Fathers’ characteristics		
Fathers’ age	11.1905 (13.967)	11.320 (13.984)
Fathers’ age squared	− 0.125 (0.154)	− 0.126 (0.154)
Fathers’ ethnicity—Irish		
Fathers’ ethnicity non-Irish	− 138.492*** (44.7949)	− 138.896*** (44.824)
Fathers’ highest educational attainment		
Fathers have primary education or none		
Fathers have secondary level education	107.785*** (31.88086)	108.205*** (31.882)
Fathers have third level education or higher	378.450*** (40.356)	379.113*** (40.369)
Fathers’ health status		
Fathers have a chronic illness or disability	− 3.864(54.217)	− 3.456 (54.239)
Fathers not in paid work		
Fathers in part-time paid work	135.542 (107.626)	136.734 (107.876)
Fathers in full-time paid work	361.879*** (44.271)	362.160*** (44.326)
Household level characteristics		
Divorced/separated/single		
Cohabitating with spouse/partner	− 13.544 ( 35.011)	− 14.274 (34.995)
Number of children		
2	57.888 (39.908)	58.861 (39.779)
3	65.316 (39.812)	66.021* (39.676)
4	135.651*** (43.813)	136.361*** (43.663)
5	193.163** (74.753)	195.052*** (74.707)
Household members who do not have a chronic illness/disability		
Household members do have a chronic illness/disability	− 92.372 (57.098)	− 91.839 (57.082)
Household living in urban area		
Household living in a rural area	− 140.634*** (28.092)	− 140.411*** (28.075)
Household have no regular access to public transport		
Household have regular access to public transport	47.342** (23.115)	47.321** (23.120)
Household living in non-rented accommodation		
Household lives in rented accommodation	− 229.381*** (33.231)	− 229.313*** (33.257)
R-Squared	0.1511	0.1513
F-Statistic	29.88	27.94
N	3759	3759

***Denotes significant at 1%, **denotes significant at 5%, *denotes significant at 10%

Sampling weights were applied due to the presence of heteroscedasticity. Clustered standard error results are presented in parenthesis

### Results of probit regression for standard of living indicator model

Table 2 presents marginal effects (ME) for the probability of households being more likely to experience difficulty making ends meet. The marginal effects results are included in this paper for ease of interpretation, as the coefficient estimates presented in Table 3 which will be used to calculate the extra cost of disability (presented in Table 4) cannot be interpreted directly. Column 1 in Table 2 shows no significant association between households who have a child with a disability, ceteris paribus, experiencing difficulty making ends meet increases by (ME = 0.027). Households where the mother or father have a chronic illness/disability are more likely to experience difficulty making ends meet by (ME = 0.058***) (ME = 0.037*), respectively. Column 2 shows no significant association was found between households who have a child severely limited in daily activities experiencing difficulty making ends meet (ME = 0.119), *ceteris paribus*, in comparison to households that do not have a child with a chronic illness/disability. Households who have a child limited somewhat in their daily activities by their condition also have an increased probability of experiencing difficulties making ends meet (ME = 0.050*). However, no significant association was found between households who have a child with a chronic illness/disability but is not limited in their daily activities experiencing difficulties making ends meet by (ME = 0.008).

**Table 2:** Binary probit model using standard of living indicator—does your household experience difficulty making ends meet average marginal effects (non-weighted)

Coefficients	Model 1	Model 2
Children with a disability	0.027 (0.019)	
Children have a severe limitation in daily activities		0.119 (0.097)
Children have some limitation in daily activities		0.050* (0.029)
Children have no limitation in daily activities as a results of their condition		0.008 (0.024)
Weekly household income residual	− 0.0002*** (0.00002)	− 0.0002*** (0.00002)
Weekly income squared residual	− 1.23e− 08 (2.94e−08)	− 1.27e-08 (2.95e-08)
Number of children		
2	0.010 (0.027)	0.008 (0.027)
3	− 0.002 (0.030)	− .0029248 (0.030)
4	− 0.016 (0.035)	− 0.018 (0.035)
5	− 0.031 (0.044)	− 0.034 (0.044)
Mothers’ ethnicity	0.037 (0.023)	0.037 (0.023)
Mothers’ highest educational attainment		
Mothers have a secondary level education	− 0.176*** (0.055)	− 0.175*** (0.055)
Mothers have a third level education or higher	− 0.234*** (0.056)	− 0.232*** (0.056)
Mothers have a chronic illness/disability	0.058*** (0.017)	0.059*** (0.017)
Fathers’ ethnicity	0.042* (0.023)	0.043* (0.023)
Fathers’ highest educational attainment		
Fathers have a secondary level education	− 0.077** (0.039)	− 0.077** (0.038)
Fathers have a third level education or higher	− 0.133*** (0.040)	− 0.134*** (0.040)
Fathers have a chronic illness/ disability	0.037* (0.020)	.0362* (0.020)
Marital status	− 0.065*** (0.025)	− 0.064** (0.025)
Household members who have a chronic illness/disability	0.060 (.0439)	0.059 (0.044)
Household number	0.043*** (0.009)	0.044 (0.009)
Household living in rural area	0.026** (0.012)	.0257603** (0.012)
Household live in rented accommodation	0.125*** 0.020)	0.125*** (0.020)
AIC	2769.839	2771.606
BIC	− 265.083	− 250.853
Pseudo R^2^	0.1361	0.1368
N	3,759	3,759

***Denotes significant at 1%

**Denotes significant at 5%, * denotes significant at 10%

**Table 3:** Binary probit model using standard of living indicator—does your household experience difficulty making ends meet (non-weighted)

Coefficients	Model 1	Model 2
Children with a disability	0.136 (0.093)	
Children have a severe limitation in daily activities		0.595 (0.486)
Children have some limitation in daily activities		0.251* (0.146)
Children have no limitation in daily activities as a results of their condition		0.039 (0.121)
Weekly household income residual	− 0.001*** (.00009)	− 0.001*** (0.00009)
Weekly income squared residual	− 6.13e-08 (1.47e-07)	− 6.33e-08 (1.47e-07)
Number of children		
2	0.048 (0.135)	0.041 (0.135)
3	− 0.008 (0.150)	− 0.015 (0.151)
4	− 0.083 (0.1781)	− 0.094 (0.179)
5	− 0.164 (0.240)	− 0.182 (0.241)
Mothers’ ethnicity	0.183 (0.116)	0.183 (0.116)
Mothers’ highest educational attainment		
Mothers’ have a secondary level education	− 0.625*** (0.169)	-0.623*** (0.169)
Mothers’ have a third level education or higher	− 0.913*** (0.175)	− 0.906*** (0.176)
Mothers’ have a chronic illness/ disability	0.287*** (0.086)	0.293*** (0.086)
Fathers’ ethnicity	0.210* (0.113)	0.214* (0.113)
Fathers’ highest educational attainment		
Fathers have a secondary level education	− 0.308** (0.140)	− 0.309** (0.140)
Fathers have a third level education or higher	− 0.596*** (0.150)	− 0.599*** (0.150)
Fathers have a chronic illness/ disability	0.184* (0.099)	0.181* (0.100)
Marital status	− 0.324*** (0.126)	− 0.321** (0.126)
Household members who have a chronic illness/disability	0.301 (.0220)	0.295 (0.219)
Household number	0.217*** (− .046)	0.219*** (0.046)
Household living in rural area	0.129** (0.058)	0.129** (0.058)
Household live in rented accommodation	0.625*** (0.101)	0.624*** (0.101)
AIC	2769.839	2771.606
BIC	− 265.083	− 250.853
Pseudo R^2^	0.1361	0.1361
N	3,759	3,759

***Denotes significant at 1%,

**Denotes significant at 5%

*Denotes significant at 10% in addition to clustered standard errors

**Table 4:** Estimates of the extra cost of child chronic illness/disability

	Income Quintile 1	Income Quintile 2	Income Quintile 3	Income Quintile 4	Income Quintile 5
Household that has a child with a chronic illness/disability	Mean income = € 445.04 % of income = 29% Extra cost = €128.96	Mean income = € 730.96 % of income = 17% Extra cost = €124.81	Mean income = € 954.47 % of income = 13% Extra cost = €121.75	Mean income = € 1219.70 % of income = 10% Extra cost = €118.31	Mean income = € 1850.92 % of income = 6% Extra cost = €110.85
Household that has a child who is hampered severely on a daily basis^a^	Mean income = € 455.41 % of income = 124% Extra cost = €562.57	Mean income = € 745.35 % of income = 73% Extra cost = €543.70	Mean income = € 910.77 % of income = 59% Extra cost = 533.49	Mean income = € 1261.54 % of income = 41% Extra cost = €513.06	Mean income = € 2204.49 % of income = 21% Extra cost = €465.18
Household that has a child who is hampered somewhat on a daily basis	Mean income = € 448.39 % of income = 53% Extra cost = €237.52	Mean income = € 722.23 % of income = 32% Extra cost = €229.97	Mean income = € 941.81 % of income = 24% Extra cost = €224.26	Mean income = € 1225.09 % of income = 18% Extra cost = €217.30	Mean income = € 1778.47 % of income = 12% Extra cost = €204.87
Household that has a child who is not hampered on a daily basis	Mean income = € 440.8249 % of income = 10% Extra cost = €43.44	Mean income = € 735.46 % of income = 5% Extra cost = €35.68	Mean income = € 964.30 % of income = 4% Extra cost = €34.76	Mean income = € 1213.29 % of income = 3% Extra cost = €33.81	Mean income = € 1861.85 % of income = 2% Extra cost = €31.56

^a^It is important to note under the Department of Social Welfare Payments scheme only households who have a child very severely affected on a daily basis receives domiciliary care payment allowance of €309.50 per month which is not means test. Households who do qualify for this payment can then apply for a carers allowance/benefit payment which is means tested and a respite care grant. (Department of Social and Family Affairs, 2014) [47]

### Results of extra cost of child chronic illness/disability

Table 4 presents the estimates of the extra cost of child chronic illness/disability across income quintiles, which shows that the extra cost of disability rises with severity. The coefficient estimates from Table 3 and the mean income from each income quintile for each disability category were used to calculate the extra cost of disability.

The households that have a child with a chronic illness/disability and earn a mean income of €445.04 per week in income quintile 1, require an extra €129 (rounded up) to achieve the same standard of living as households with the same characteristics, but do not have a child with a disability. When the extra cost of disability is examined for all households in income quintile 1 based on degree of severity, the results show that the extra cost of disability is €563 per week for households that have a child severely hampered on a daily basis. Households that have a child hampered somewhat need €237 more per week to achieve the same standard of living, while households that have a child not hampered on a daily basis incur an extra cost of disability €43 per week. Those in the highest income quintile have extra costs of €465, €205, and €31 respectively, depending on how the child disability variable in specified.

It is important when interpreting these results to bear in mind the pattern of significance in the regression analyses though. That is, while additional costs can be estimated for severity and income groups the ordering of which may seem plausible, coefficients were not always statistically significant. Caution is therefore warranted. Sensitivity analyses reported in Tables S13–S14 show that analyses based on weighted and unweighted analyses and based on the alternative modelling approach [44] do not provide statistically significant coefficients in the probit models, though as it was not possible to bootstrap the weighted results, particular care is therefore warranted with these results and no results are provided for the weighted version. In brief, an income penalty is detected in the OLS equation and an impact of disability at the 10% level detected in respect of the somewhat disabled group.

## Discussion

The impact of child chronic illness/disability on family income, ability to sustain employment and predispositions to poverty is a pertinent issue for policymakers and presents greater challenges to address in light of economic uncertainty due to COVID-19 and lockdowns. Current social welfare payments in Ireland provide a monthly Domiciliary Care Allowance (DCA) payment of €309.50, however, the eligibility criteria is very strict. “Eligible children from birth to the age of 16 who are living at home and who have a severe disability requiring continual or continuous care and attention which is substantially in excess of that normally required by a child of the same age may qualify for Domiciliary Care Allowance. The condition must be likely to last at least one year” [47].

The results presented above illustrate the income handicap associated with raising a child with a disability. Of particular interest is the insight into the variation in estimates based on which child disability variable is used. A €96.35 reduction in weekly household income for those who have a child with a chronic illness/disability poses a considerable challenge to the economic burden faced by families raising a child with a disability. In terms of the social welfare disability-related supports available to these families, only households who have a child severely hampered on a daily basis qualify for domiciliary care payments and possibly carers payment which are means tested. It is important to note that the estimates for having a child with a severe limitation in daily activities takes these disability-related payments into consideration as part of its reported household income when calculating entitlements. Nevertheless, an important finding of policy concern is the statistically significant reductions of €116.68 and €78.11 per week for households who have a child hampered somewhat or those whose child has no limitations in daily activities, respectively. These findings are plausible, reflecting the potential “trap” faced by families whose children are disabled but not disabled enough to qualify for specific transfer payments that may help mitigate loss of income. This suggests policy makers may wish to consider either adjusting thresholds to entitlements and/or mitigating income loss by better accommodating parents who can and want to earn additional income by supporting the provision of flexible working arrangements and suitable special needs childcare. A strength of the analysis is that parents in the survey had no incentive to “game” their responses, i.e. overstate the degree of disability. Though, an objective measure of need rather than self-reported parental assessments would strengthen policy recommendations. In terms of the previous literature on this topic, it is difficult to make direct comparisons as the indirect cost i.e. loss in income, has tended to be measured either through labour supply models or cost of illness studies.

In relation to the probit models which were used to calculate the extra cost of disability, it is important to remember that only households with a child hampered somewhat had marginal effects statistically significant at a 10% level (ME = 0.251*). While it may seem disappointing that the results did not provide a clearer signal of the role of disability on SoL, not only must the limitations of the study be borne in mind, but so too must potential explanations for the findings other than study limitations. For example, expectations on the part of respondents may play an important role in framing responses to subjective questions such as SoL. Parents with disabled children that is, may have adapted their expectations of what constitutes an acceptable SoL making the measure less sensitive than might be hoped. The constraints placed on family life and without appropriate supports from extended family or suitable childcare, may see parents reconcile themselves to conditions families with non-disabled children do not. As we do not observe the length of time, the child has had their condition in the survey, the study is limited and may in consequence underestimate the magnitude of the conversion handicap effects sought in the SoL approach.

The findings presented in relation to the extra cost of child chronic illness/disability provide empirical insights into the inequality that arises as a result of variations in need between households with and without a child with a chronic illness/disability, and the required income needed to compensate to achieve the same standard of living. If the results of the probit models are taken at face value the coefficients that were used in calculating the extra cost of disability show a relationship in which extra costs rise with severity, which as noted are intuitively plausible. The extra cost findings are consistent with the broad evidence from previous studies [27, 34, 48–50] in terms of households who have an individual with a chronic illness/disability experiencing a lower standard of living as a result of the extra cost of disability, which increases with the severity level of disability. In relation to the four previous studies to estimate the extra cost of child disability [8, 10–12], the extra cost of disability as a percentage of income ranged from 10 to 47%. In this study, the extra cost of disability ranged from 3 to 61% of family income depending on the severity of the child’s condition and which income quintile the family’s household income fell into. This level of detail provides policymakers with greater information concerning the financial hardship endured by families, particularly those in lower income quintiles. In addition, the extra cost of disability does not imply that all needs are being met and the issue of unmet needs warrants further examination.

While most of the previous studies conducted have focused on the extra cost of adult disability, it is not possible to make direct comparisons between the estimates provided in this paper and previous studies on the extra cost of adult disability given the distinct differences between child and adult disabilities. Adult disability can have a substantial impact on household income as the only source of income may be confined to a modest disability-related payment. Thus, any expenditure on disability-related needs may be subject to significant income constraints. In addition, household composition and needs for adults with a chronic illness/disability may vary in comparison to households raising a child with a chronic illness/disability. These differences may in part explain why not all the coefficients for the child disability variables were statistically significant in the models. (With children, parents can to a degree mitigate the impact of disability). A similar trend was evident in Mont and Cuong [12] where the sample was aged 5 years and older. No coefficient results were presented in Burchardt and Zaidi [8]. Nevertheless, it is worth noting that from the adult disability studies not all the disability variable coefficients were statistically significant, such that those with severe disabilities were more likely to have statistically significant coefficients in contrast to those less severely affected by their disability. Given the varying dynamics that influence the extra cost of disability between adult and child chronic illness/disability, this paper highlights the need to examine the extra cost of child chronic illness/disability as a distinct cohort.

The data used in this paper provide baseline estimates and limits the current interpretation of the impact of COVID-19. It is important to consider evidence on the impact of COVID-19 which may exasperate an already challenging situation for families who have a child or children with a disability. The disruption to disability, health, educational and social services, routine, childcare provision and employment either through adjusting to remote working or job losses places an even greater strain upon caregivers [51, 52]. Projections on the impact of COVID-19 on child poverty estimated an additional 142 million children would be living in monetary poverty by the end of 2020 [53]. Darmody et al. [54] provide a useful insight into the economic and work-related challenges associated with COVID-19 in Ireland, but there is no specific information in relation to the economic impact for families caring for a child or children with a chronic illness/disability. A UK study which examined the impact of COVID-19 on parental stress and support needs found that within the total sample (5000 parents/carers) work was cited as the highest source of stress amongst 53.1% of parents. However, parents/carers with a child with special educational needs or a neurodevelopmental condition reported feeling more stressed about all of the stressors listed (child’s wellbeing, friends and family outside the home, their child’s education and their child’s behaviour) apart from work (50.9%), in comparison to parents with a child without special educational needs or a neurodevelopmental condition [55]. This may be explained by the fact parents/carers are less likely to be in employment.

The estimated cost of disability presented in this paper highlights that DCA payments do not appear to adequately compensate the financial burden incurred by families in the somewhat limited category. These households receive no financial assistance, yet they incur a substantial financial burden accounting for 12–53% and 2–10% of their weekly income, depending on which income quintile their income falls into. The implications of these findings suggest that a tiered approach to disability support payments which encompass broader criteria for inclusion based on varying severity levels be introduced to alleviate the financial hardship and compromised economic wellbeing of families affected, assuming society wishes to mitigate this inequality. Had the analysis just focused on calculating the extra cost of disability based on the variable for having a child with a disability, it would have misrepresented the economic impact incurred by families. Future research would benefit from focusing on specific chronic illnesses or disabilities given the heterogeneity that exists amongst children with chronic illnesses/disabilities.

## Limitations

This paper has a number of limitations which should be taken into consideration when reviewing the findings. First, the analysis is based on data collected during 2007–2008, because this was the most recent dataset available for this age group at the time of analysis. While public policy would benefit from more recent data to inform policy and practice, the lack of timely datasets to analyze the standard of living has also been an issue in previously published standard of living approach studies [26–28]. Second, the estimates are based on cross-sectional data so that the results could be compared to most of the previous studies which used cross-sectional data. Therefore, the focus is on the association of the relationships, as opposed to drawing casual inferences. Thirdly, due to the nature of available datasets in Ireland it was only possible to estimate the cost of disability for the study children whose ages range from 8–10 years of age. It is important to acknowledge that little is known about how the cost of disability varies amongst different age groups of children, apart from one study which segregated children by different age groups for their analysis [11]. Another limitation worth acknowledging is the fairly low R-squared results in the models, despite a large number of predictors and large sample size. Future research would benefit from the availability of variables on the extent of unmet health, social and educational needs experienced by the child. The level of unmet needs may help explain parents’ ability to sustain employment and the extra cost of disability they incur from paying privately for services and supports that are not available in the public system. Furthermore, the lack of precise information about siblings’ disability status prevented a more detailed analysis of estimating the cost of childhood disability based on the number of children with a chronic illness/disability in the household. Nevertheless, the study was able to control for whether a household member other than a parent had a chronic illness/disability which affected the study child.

The measure of disability used in this analysis provided insight into the severity of a multitude of different chronic illnesses/disability for children. It is important, however, to stress that this meaning is dependent upon the condition the child has and needs to be understood in that context. Thus, the nature and impact of disability with respect to asthma (say) is very different to that of autism (say) as will the impact. This heterogeneity cannot be addressed with the data available here. Similarly, while the use of a subjective indicator as a measure of standard of living has the advantage that it reflects the current situation of the household, an issue which may not be captured as accurately based on ownership of household items which may have been purchased before the onset of the child’s condition, it is not without its limitations. As noted these include the possibility for households to adapt to circumstances and revise their assessment of their SoL. The lack of an objective measure even if only for robustness checks is thus an issue. Interestingly, a study which used both a subjective and objective indicator which found that in the 27 countries analysed, only 8 of the countries had a higher cost of disability using the subjective indicator, which they suggested was “downward adaptation of the expectations of households with disabled people that allows for them to make ends meet with lower increases in household income to compensate for the higher needs of disabled people” [34].

## Conclusion

Despite the limitations set out above, this paper has provided insights into the income and conversion handicaps experienced by families raising a child with a disability in Ireland. In particular it has given prominence to both the ‘horizontal equity’ and ‘vertical equity’ issues that arises as a result of lack of income and variations in need between households with and without a child with a chronic illness/disability based on the degree of severity to which the child is hampered daily. Furthermore, it has highlighted the distinct differences that influence the cost of adult and child disabilities and points out the need to examine the extra cost of child chronic illness/disability as a distinct cohort. A strength of the study is that it has acknowledged the issue of potential endogeneity and attempted to address it, unlike previous studies. It is fully acknowledged though that this is an area in which further research effort could be usefully expended.

The findings suggest that policymakers may need to adopt a new perspective when it comes to addressing the cost of child disability and its associated hardship. Current thinking is focused on cost cutting and deems it an additional burden that the exchequer cannot afford to address. However, this myopic approach of cost cutting, which can translate to cost shifting has substantial implications for the health and economic wellbeing of families raising a child with a chronic illness/disability. More innovative policies are required to implement appropriate timely access to health and social care services and flexi parental employment, which in turn requires the provision of adequate access to high-quality educational and care facilities.

## Supplementary Information

Below is the link to the electronic supplementary material.

Supplementary file1 (DOCX 73 kb)

## Footnotes

1 Nelson [18] presents a comprehensive overview of the history of equivalisation and diverging arguments concerning what is “household welfare”. See also Burchardt and Zaidi [8] who provide a helpful overview of other approaches to estimating equivalence scales.

2 See Tibble [22] and Berthoud et al.’s [17] for a critique of the various methodologies used to measure the additional costs of disability.

3 8417 of the study children were aged 9, while 62 of the study children were aged 8 years and 75 aged 10 years respectively.

4 The Household Budget Survey (HBS) does contain information on whether a child aged under 15 years of age has a chronic illness, however the question is not based on ICD-10 criteria and does not measure the severity to which the child is affected. Furthermore, there is no information on household income, a measure which underpins the analysis. EU-SILC data does not contain information about the disability status of the child. See [31] for further details.

5 There were 620 missing values in the household income variable.

6 To proceed with an ordered logit model, the model had to be tested to see if the parallel regression assumption, otherwise known as the proportional odds assumption held or was violated. This means that each of the outcome responses must carry equal weighting or the same proportional odds of being reported based on the coefficients in the model. If this assumption is violated, it would require using different models for each category of the outcome groups. The omodel and Brant tests performed showed that the parallel regression assumption was violated by the income variables and maternal disability variables. While generalized ordered logit models are considered a solution to correct for the violation of the proportional odds assumption, a probit model was considered the most parsimonious model to use for two reasons outlined in the econometric model specifications section. A binary variable was constructed for the SoL indicator as this approach is accepted within the literature. For instance, Zaidi and Burchardt [21] also used another SoL indicator based on a binary variable for whether a household had savings or not. The variable had to be converted from an ordered categorical variable to a binary variable for the econometrics analysis. The variable was dichotomised as 1 = great difficulty/with difficulty/ with some difficulty and 0 = fairly easily/easily/very easily.

7 There is no definitive definition of child disability in Ireland, therefore, the terms chronic illness/disability will be used in this paper. Discrepancies have emerged in child disability prevalence rates cited for Ireland due to variations in definitions and methodologies [36].

8 Information on chronic illness or disability amongst other household members is accounted for in the. following variable – “Does anyone in your household CURRENTLY have any chronic illness or. disability which adversely affects the Study Child?” followed by a breakdown of whether they are a. parent/sibling/relative/non-relative. In addition, there are specific chronic illness/disability variables for. the primary and secondary carers.

9 See Table S10 and S11 on testing income specifications in the supplemental section.

10 For further details on generalized ordered logit models ‘gologit2’ see [42, 45].

## Declarations

### Availability of data and material:

The Growing Up in Ireland Dataset can be accessed through the Irish Social Sciences Data Archive.
