# Supplementary material for: Income and conversion handicaps: estimating the impact of child chronic illness/disability on family income and the extra cost of child chronic illness/child disability in Ireland using a standard of living approach
Source: Eur J Health Econ. 2021 Sep 9;23(3):467–83. doi: 10.1007/s10198-021-01371-4 (PMC8426335; doi:10.1007/s10198-021-01371-4)
Supplement: Supplementary file 1 — Supplementary file1 (DOCX 73 kb) [file 10198_2021_1371_MOESM1_ESM.docx]

**Supplemental Material**

Income and Conversion Handicaps: Estimating The Impact of Child Chronic Illness/Disability on Family Income and the Extra Cost of Child Chronic Illness/Disability in Ireland Using a Standard of Living Approach

The European Journal of Health Economics

**Table S1: Prevalence of Any On-Going Chronic Physical or Mental Health**

**Problem, Illness or Disability in the GUI Nine Year Old Child Cohort**

|  | **Disability Status** | | | | |  | |
| --- | --- | --- | --- | --- | --- | --- | --- |
|  | **Child without a chronic illness/disability** | | **Child with a chronic illness/disability** | | | **Total** | |
|  | **N** | **%** | | **N** | **%** | **N** | **%** |
| Boys | 3,666 | 47.67 | | 489 | 56.66 | 4,155 | 48.57 |
| Girls | 4,025 | 52.33 | | 374 | 43.34 | 4,399 | 51.43 |
| Total | 7,691 | 100.00 | | 863 | 100.00 | 8,554 | 100.00 |
| Chi- square test χ^2^ = 25.1423 , <0.000 | | | | | | | |

Table S2: Extent to Which Child is Hampered Daily in His/ Her Activities by Disability

|  | **Severity of Disability** | | | | | | | | | |
| --- | --- | --- | --- | --- | --- | --- | --- | --- | --- | --- |
|  | **Child severely hampered** | | | **Child hampered to some extent** | | | **Child not hampered** | | **Total** | |
|  | **N** | | **%** | **N** | | **%** | **N** | **%** | **N** | **%** |
| Boys | 29 | | 5.93 | 166 | | 33.95 | 294 | 60.12 | 489 | 100.00 |
| Girls | 18 | | 4.81 | 140 | | 37.43 | 216 | 57.75 | 374 | 100.00 |
| Total | 47 | | 5.45 | 306 | | 35.45 | 510 | 59.10 | 863 | 100.00 |
| Chi-square test | | χ^2^=3.2608, <0.071 | | | χ^2^= 4.0910, < 0.043 | | χ^2^=17.8735, <0.000 | | | |

Table S3: Prevalence of Child’s Current Chronic Illness 1 Based on the International Classification of Diseases 10

|  | **Boys** | | **Girls** | | **Total** | |
| --- | --- | --- | --- | --- | --- | --- |
|  | **N** | **%** | **n** | **%** | **n** | **%** |
| Diseases of the respiratory system | 232 | 47.44 | 175 | 46.79 | 407 | 47.16 |
| Diseases of the nervous system | 21 | 4.29 | 17 | 4.55 | 38 | 4.40 |
| Congenital malformations, deformations and chromosomal abnormalities | 13 | 2.66 | 16 | 4.28 | 29 | 3.36 |
| Diseases of the skin and subcutaneous tissue | 14 | 2.86 | 18 | 4.81 | 32 | 3.71 |
| Endocrine, nutritional and metabolic diseases | 13 | 2.66 | 11 | 2.94 | 24 | 2.78 |
| Diseases of the genitourinary system | 9 | 1.84 | 23 | 6.15 | 32 | 3.71 |
| Diseases of the digestive system | 13 | 2.66 | 22 | 5.88 | 35 | 4.06 |
| Diseases of the circulatory system | 14 | 2.86 | 10 | 2.67 | 24 | 2.78 |
| Diseases of the musculoskeletal system and connective tissue | 12 | 2.45 | 14 | 3.74 | 26 | 3.01 |
| Diseases of the ear and mastoid process | 23 | 4.70 | 14 | 3.74 | 37 | 4.29 |
| Diseases of the eye and adnexa | 10 | 2.04 | 5 | 1.34 | 15 | 1.74 |
| Neoplasms | 3 | 0.61 | - | - | 3 | 0.35 |
| Other diseases not classified elsewhere | 4 | 0.82 | 9 | 2.41 | 13 | 1.51 |
| Certain infectious and parasitic issues | 3 | 0.61 | 1 | 0.27 | 4 | 0.46 |
| Mental, behavioural disorders | 104 | 21.27 | 36 | 9.63 | 140 | 16.22 |
| Diseases of the blood and blood forming organs and certain disorders of the immune system | 1 | 0.20 | 3 | 0.80 | 4 | 0.46 |
| Total | 489 | 100 | 374 | 100 | 863 | 100 |

Table S4: Prevalence of Child’s Current Chronic Illness 2 Based on the International Classification of Diseases 10

|  | **Boys** | | **Girls** | | **Total** | |
| --- | --- | --- | --- | --- | --- | --- |
|  | **N** | **%** | **n** | **%** | **n** | **%** |
| Diseases of the respiratory system | 6 | 14 | 5 | 24 | 11 | 17 |
| Congenital malformations, deformations and chromosomal abnormalities | 1 | 2 | - | - | 1 | 1 |
| Diseases of the skin and subcutaneous tissue | 3 | 7 | 4 | 19 | 7 | 11 |
| Diseases of the genitourinary system | - | - | 2 | 9 | 2 | 3 |
| Diseases of the digestive system | 3 | 7 | 2 | 9 | 5 | 8 |
| Diseases of the circulatory system | 2 | 5 | 1 | 5 | 3 | 5 |
| Diseases of the ear and mastoid process | 3 | 7 | - | - | 3 | 5 |
| Diseases of the eye and adnexa | 1 | 2 | - | - | 1 | 1 |
| Other diseases not classified elsewhere | 1 | 2 | 1 | 5 | 2 | 3 |
| Mental, behavioural disorders | 24 | 54 | 6 | 29 | 30 | 46 |
| Total | 44 | 100 | 21 | 100 | 65 | 100 |

Table S5: Prevalence of Child’s Current Chronic Illness 3 Based on the International Classification of Diseases 10

|  | **Boys** | | **Girls** | | **Total** | |
| --- | --- | --- | --- | --- | --- | --- |
|  | **N** | **%** | **n** | **%** | **n** | **%** |
| Diseases of the nervous system | 1 | 11 | - | - | 1 | 7.6 |
| Diseases of the skin and subcutaneous tissue | - | - | 1 | 25 | 1 | 7.6 |
| Diseases of the ear and mastoid process | - | - | 1 | 25 | 1 | 7.6 |
| Mental, behavioural disorders | 8 | 89 | 2 | 50 | 10 | 77.2 |
| Total | 9 | 100 | 4 | 100 | 13 | 100 |

Table S6: Characteristics of the Study Sample (weighted)

| **Variables** | **Household with a Non-Disabled Child**  **N= 7,691** | **Household with a Child with a Disability**  **N= 863** | **Design-based F** |
| --- | --- | --- | --- |
| **Mothers’ characteristics** |  |  |  |
| Mothers’ participating in the labour force | 57.38% | 49.01% | 0.0002 |
| Mothers’ ethnicity-non-Irish | 9.12% | 7.67% | 0.2636 |
| Mothers’ have a disability | 12.23% | 23.01% | 0.0000 |
| Mothers’ are depressed | 29.75% | 26.66% | 0.2860 |
| Mothers’ highest educational attainment - primary education or less | 6.09% | 8.68% | 0.0452 |
| Mothers’ highest educational attainment- secondary level education | 60.6% | 62.77% | 0.3205 |
| Mothers’ highest educational attainment -third level qualification or higher | 33.31% | 28.55% | 0.0166 |
| **Fathers’ characteristics** |  |  |  |
| Fathers’ participating in labour force | 95.48% | 93.74% | 0.1812 |
| Fathers’ ethnicity- non-Irish | 8.54% | 9.03% | 0.7176 |
| Fathers’ have a disability | 8.63% | 12.85% | 0.0035 |
| Fathers’ highest educational attainment- primary education or less | 6.59% | 7.12% | 0.7389 |
| Fathers’ highest educational attainment- secondary level education | 56.71% | 55.08% | 0.5040 |
| Fathers’ highest educational attainment- third level education or higher | 36.7% | 37.81% | 0.6309 |
| **Household Characteristics** |  |  |  |
| Currently married/living with partner | 77.4% | 69.73% | 0.0003 |
| Divorced/separated/single | 22.6% | 30.27% | 0.0003 |
| Other household members with a chronic illness/disability | 2.06% | 3.7% | 0.0173 |
| Equivalised household income in quintiles |  |  |  |
| Lowest | 19.47% | 23.57% | 0.0493 |
| 2^nd^ | 19.63% | 23.54% | 0.0448 |
| 3^rd^ | 20.14% | 19.63% | 0.7765 |
| 4^th^ | 20.38% | 16.26% | 0.0167 |
| Highest | 20.38% | 17% | 0.0411 |
| Household have full medical card coverage | 26.47% | 40.44% | 0.0000 |
| Household have Doctor only medical card coverage | 2.77% | 3.90% | 0.1527 |
| Household have no medical card coverage | 70.76% | 55.66% | 0.0000 |
| Household have full private health insurance | 45.96% | 40.65% | 0.0143 |
| Household have partial private health insurance | 2.77% | 3.90% | 0.1527 |
| Household have no private health insurance | 70.76% | 55.66% | 0.0000 |
| Household lives in rural area | 55.74% | 51.12% | 0.0400 |
| Household lives in rented accommodation | 19.45% | 28.97% | 0.0000 |

Note: The characteristics of the study sample (unweighted) are provided in Table S7 in the Appendix.

Table S7: Characteristics of the Study Sample (unweighted)

| **Variables** | **Household with a Non-Disabled Child**  **N= 7,691** | **Household with a Child with a Disability**  **N= 863** | **Chi-Square**  **χ^2^** |
| --- | --- | --- | --- |
| **Mothers’ characteristics** |  |  |  |
| Mothers’ participating in the labour force | 59.59% | 53.37% | 0.000 |
| Mothers’ ethnicity-non-Irish | 9.00% | 8.79% | 0.842 |
| Mothers’ have a disability | 11.84% | 23.08% | 0.000 |
| Mothers’ are depressed | 29.39% | 28.57% | 0.732 |
| Mothers’ highest educational attainment - primary education or less | 3.11% | 5.03 % | 0.003 |
| Mothers’ highest educational attainment- secondary level education | 45.41% | 49.94% | 0.012 |
| Mothers’ highest educational attainment -third level qualification or higher | 51.49% | 45.03% | 0.000 |
| **Fathers’ characteristics** |  |  |  |
| Fathers’ participating in labour force | 97.19% | 96.43% | 0.267 |
| Fathers’ ethnicity-  non-Irish | 9.12% | 9.64% | 0.656 |
| Fathers’ have a disability | 7.84% | 12.44% | 0.000 |
| Fathers’ highest educational attainment- primary education or less | 4.26 % | 4.12% | 0.857 |
| Fathers’ highest educational attainment- secondary level education | 51.11% | 50.59 % | 0.795 |
| Fathers’ highest educational attainment- third level education or higher | 44.62% | 45.29% | 0.738 |
| **Household Characteristics** |  |  |  |
| Currently married/living with partner | 83.19% | 76.94% | 0.000 |
| Divorced/separated/single | 16.81% | 23.06% | 0.000 |
| Other household members with a chronic illness/disability | 2.01% | 3.78% | 0.001 |
| Equivalised household income in quintiles |  |  |  |
| Lowest | 12.93% | 16.27% | 0.008 |
| 2^nd^ | 17.03% | 19.75% | 0.053 |
| 3^rd^ | 19.81% | 20.99% | 0.425 |
| 4^th^ | 23.23% | 19.63% | 0.021 |
| Highest | 26.99% | 23.35% | 0.027 |
| Household have full medical card coverage | 18.05% | 29.32% | 0.000 |
| Household have Doctor only medical card coverage | 2.33% | 3.59% | 0.023 |
| Household have no medical card coverage | 79.62% | 67.09% | 0.000 |
| Household have full private health insurance | 56.02% | 51.45% | 0.010 |
| Household have partial private health insurance | 9.67% | 7.30% | 0.024 |
| Household have no private health insurance | 34.32% | 41.25% | 0.000 |
| Household lives in rural area | 54.94% | 50.70% | 0.018 |
| Household lives in rented accommodation | 14.72% | 20.39% | 0.000 |

Table S8: Description of GUI Variables Used in Weekly Income Models

| **Variable** | **Variable Description** |
| --- | --- |
| ***Dependent variables*** | |
| Weekly household income (linear) | The dependent variable is a continuous variable representing weekly household income |
| ***Main Independent variables*** | |
| Children with a chronic illness/disability | Indicator variable taking a value of 1 if the nine year old child has a chronic illness/disability and 0 otherwise |
| Children have a severe limitation in daily activities | Indicator variable taking a value of 1 if the nine year old child has a severe limitation in daily activities and 0 otherwise |
| Children have some limitation in daily activities | Indicator variable taking a value of 1 if the nine year old child has some limitation in daily activities and 0 otherwise |
| Children have no limitation in daily activities as a result of their condition | Indicator variable taking a value of 1 if nine year old child has no limitation in daily activities as a result of their condition and 0 otherwise |
| ***Explanatory variables*** | |
| Mothers’ age | Mother’s age^[[1]](#footnote-1)^ |
| Mothers’ ethnicity | Indicator variable taking a value of 1 if the mother is non- Irish and 0 otherwise |
| Mothers’ highest educational attainment | Primary education or none- indicator variable taking a value of 1 if the mother has a primary education and 0 otherwise; Secondary level- indicator variable taking a value of 1 if the mother has a secondary level education and 0 otherwise; third level or higher- indicator variable taking a value of 1 if the mother has a third level education or higher and 0 otherwise |
| Mothers’ disability status | Indicator variable taking a value of 1 if the mother has a chronic illness or disability and 0 otherwise |
| Mothers’ depression status | Indicator variable taking a value of 1 if the mother is depressed based on CES-D scale and 0 otherwise |
| Mothers’ employment status | In part-time paid work takes a value of 1 and 0 if not in paid work; full-time paid work takes a value of 1 and 0 if not in paid work |
| Fathers’ age | Father’s age^[[2]](#footnote-2)^ |
| Fathers’ ethnicity | Indicator variable taking a value of 1 if the father is non- Irish and 0 otherwise |
| Fathers’ highest educational attainment | Primary education or none- indicator variable taking a value of 1 if the father has a primary education and 0 otherwise; Secondary level- indicator variable taking a value of 1 if the father has a secondary level education and 0 otherwise; third level or higher- indicator variable taking a value of 1 if the father has a third level education or higher and 0 otherwise |
| Fathers’ disability status | Indicator variable taking a value of 1 if the father has a chronic illness or disability and 0 otherwise |
| Fathers’ employment status | In part-time paid work takes a value of 1 and 0 if not in paid work; full-time paid work takes a value of 1 and 0 if not in paid work |
| Household member with a disability | Indicator variable taking a value of 1 if a household member other than the parents or study child has a chronic illness or disability and 0 otherwise |
| Martial Status | Indicator variable taking a value of 1 if cohabitating with spouse or partner and 0 if divorced, separated or single |
| Number of children | Categorical variable where the base category is 1 child, 2 is two children, 3 is three children, 4 is four children, 5 is five children |
| Rural | Indicator variable taking a value of 1 if the household lives in an rural area and 0 otherwise |
| Regular public transport | Indicator variable taking a value of 1 if the household have regular access to public transport and 0 otherwise |
| Rented accommodation | Indicator variable taking a value of 1 if the household lives in rented accommodation and 0 otherwise |

Table S9: Description of GUI Variables Used in SoL Models

| **Variable** | **Variable Description** |
| --- | --- |
| ***Dependent variables*** | |
| Difficulty making ends meet | Indicator variable taking a value of 1 if the household experiences great difficulty/ with difficulty/ with some difficulty making ends meet concerning the household’s total monthly or weekly income and 0 otherwise |
| ***Main Independent variables*** | |
| Children with a chronic illness/disability | Indicator variable taking a value of 1 if the nine year old child has a chronic illness/disability and 0 otherwise |
| Children have a severe limitation in daily activities | Indicator variable taking a value of 1 if the nine year old child has a severe limitation in daily activities and 0 otherwise |
| Children have some limitation in daily activities | Indicator variable taking a value of 1 if the nine year old child has some limitation in daily activities and 0 otherwise |
| Children have no limitation in daily activities as a result of their condition | Indicator variable taking a value of 1 if nine year old child has no limitation in daily activities as a result of their condition and 0 otherwise |
| ***Explanatory variables*** | |
| Residuals from OLS model of household income (linear) | Continuous unequivalised weekly income variable |
| Residuals from OLS model of weekly household income squared | Continuous unequivalised annual income variable squared |
| Mothers’ ethnicity | Indicator variable taking a value of 1 if the mother is non- Irish and 0 otherwise |
| Mothers’ highest educational attainment | Primary education or none- indicator variable taking a value of 1 if the mother has a primary education and 0 otherwise; Secondary level- indicator variable taking a value of 1 if the mother has a secondary level education and 0 otherwise; third level or higher- indicator variable taking a value of 1 if the mother has a third level education or higher and 0 otherwise |
| Mothers’ disability status | Indicator variable taking a value of 1 if the mother has a chronic illness or disability and 0 otherwise |
| Mothers’ depression status | Indicator variable taking a value of 1 if the mother is depressed based on CES-D scale and 0 otherwise |
| Fathers’ ethnicity | Indicator variable taking a value of 1 if the father is non- Irish and 0 otherwise |
| Fathers’ highest educational attainment | Primary education or none- indicator variable taking a value of 1 if the father has a primary education and 0 otherwise; Secondary level- indicator variable taking a value of 1 if the father has a secondary level education and 0 otherwise; third level or higher- indicator variable taking a value of 1 if the father has a third level education or higher and 0 otherwise |
| Fathers’ disability status | Indicator variable taking a value of 1 if the father has a chronic illness or disability and 0 otherwise |
| Martial Status | Indicator variable taking a value of 1 if cohabitating with spouse or partner and 0 if divorced, separated or single |
| Household member other than parents with a chronic illness/disability | Count variable of the number of members in the household other than parents with a chronic illness/disability |
| Household number | Number of people in the household |
| Household living in rural | Indicator variable taking a value of 1 if the household lives in an rural area and 0 otherwise |
| Rented accommodation | Indicator variable taking a value of 1 if the household lives in rented accommodation and 0 otherwise |

Table S10: Testing Income Specifications for Standard of Living Indicator: Dependent Binary Variable - Whether The Household has difficulty making ends meet Including Interaction Terms Based on Child Disability

| **Coefficients** | **Model 1** | **Model 2** | **Model 3** | **Model 4** | **Model 5** |
| --- | --- | --- | --- | --- | --- |
| Children with a disability |  | 0.042  (0.063 | 0.034  (0.063) | 0.036  (-.063) |  |
| Income, linear | Iterations backed up |  | -0.001***  (0.00008) |  |  |
| Income, log |  | -.870***  (0.061) |  |  |  |
| Income, squared |  |  | 1.16e-07***  (1.15e-08) |  |  |
| Income, square root |  |  |  | -0.059***  (0.005) |  |
| Income*children with a disability |  |  |  |  | Iterations backed up |
| Number of children |  |  |  |  |  |
| 2 |  | 0.083  (0.098) | 0.075  (0.098) | 0.081  (0.098) |  |
| 3 |  | 0.143  (0.106) | 0.128  (0.108) | 0.135  (0.107) |  |
| 4 |  | 0.170  (0.131) | 0.154  (0.134) | 0.162  (0.132) |  |
| 5 |  | 0.196  (0.174) | 0.173  (0.177) | 0.190  (0.175) |  |
| Marital status |  | -0.158*  (0.081) | -0.159**  (0.080) | -0.157**  (0.080) |  |
| Mothers’ ethnicity |  | 0.070  (0.082) | 0.071  (0.082) | 0.072  (0.082) |  |
| Mothers’ secondary education |  | -0.185*  (0.108) | -0.201*  (0.108) | -0.199*  (0.108) |  |
| Mothers’ third level education |  | -0.181  (0.112) | -0.184*  (0.111) | -0.188*  (0.112) |  |
| Mothers’ have a disability |  | 0.324***  (0.061) | 0.325***  (0.061) | 0.323***  (0.061) |  |
| Fathers’ ethnicity |  | 0.140*  (0.080) | 0.128  (0.081) | 0.132*  (0.080) |  |
| Fathers’ secondary education |  | -0.151*  (0.091) | -0.158*  (0.090) | -0.157*  (0.090) |  |
| Fathers’ third level education |  | -0.274***  (0.100) | -0.257***  (0.100) | -0.267***  (0.100) |  |
| Fathers’ have a disability |  | 0.171***  (0.066) | .0180***  (0.066) | 0.179***  (0.066) |  |
| Other household member with chronic illness |  | 0.281  (0.131) | 0.278**  (0.132) | 0.278**  (.1311989) |  |
| Household living in rural area |  | 0.301***  (0.067) | -0.040 (0.041) | -0.038  (0.041) |  |
| Household live in rented accommodation |  | -0.031  (0.041) | 0.311***  (0.065) | 0.311***  (0.066) |  |
| Household number |  | 0.154***  (0.033) | 0.164***  (0.033) | 0.159***  (0.033) |  |
| Linktest |  | -.1233415*** | .0341489 ^NS^ | .085649** |  |
| AIC |  | 5495.309 | 5466.369 | 5481.821 |  |
| BIC |  | -653.700 | -675.877 | -667.189 |  |
| Pseudo R^2^ |  | 0.1320 | 0.1357 | 0.1329 |  |
| Log likelihood |  | -2727.655 | -2712.184 | -2720.910 |  |
| N |  | 6,400 | 6,400 | 6,400 |  |

***denotes significant at 1%, ** denotes significant at 5%, * denotes significant at 10%.

Results are based on probit model coefficients. Clustered standard error results are presented in parenthesis.

Table S11: Testing Income Specifications for Standard of Living Indicator: Dependent Binary Variable - Whether The Household has difficulty making ends meet Based on the Degree of Severity to Which the Child is Hampered Daily

| **Coefficients** | **Model 1** | **Model 2** | **Model 3** | **Model 4** | **Model 5** |
| --- | --- | --- | --- | --- | --- |
| Children have a severe limitation in daily activities |  | 0.244  (0.256) | 0.247  (0.260) | 0.246  (0.258) | 3.383***  (1.147) |
| Children have some limitation in daily activities |  | 0.187*  (0.096) | 0.182*  (0.096) | 0.182*  (0.096) | 1.427***  (0.293) |
| Children have no limitation in daily activities as a result of their condition |  | -0.061  (0.084) | -0.072  (0.085) | -0.070  (0.085) | 0.509**  (0.205) |
| Income, linear | Iterations backed up |  | -0.001***  (0.00008) |  |  |
| Income, log |  | -0.868***  (0.061) |  |  |  |
| Income, squared |  |  | 1.16e-07***  (1.15e-08) |  |  |
| Income, square root |  |  |  | -0.059***  (0.005) |  |
| Income* Children have a severe limitation in daily activities |  |  |  |  | -0.004***  (0.001) |
| Income* Children have some limitation in daily activities |  |  |  |  | -0.001***  (0.0003) |
| Income* Children have no limitation in daily activities as a result of their condition |  |  |  |  | -0.0006***  (0.0002) |
| Number of children |  |  |  |  |  |
| 2 |  | 0.077  (0.098) | .0690748  (.0985047) | 0.075  (0.098) | 0.090  (0.095) |
| 3 |  | 0.137  (0.107) | 0.121  (0.108) | 0.129  (0.107) | 0.177*  (0.104) |
| 4 |  | 0.160  (0.132) | 0.143  (0.134) | 0.152  (0.133) | 0.204  (0.130) |
| 5 |  | 0.181  (0.175) | 0.157  (0.177) | 0.174  (0.175) | 0.280*  (0.170) |
| Marital status |  | -0.157*  (0.081) | -0.158**  (0.080) | -0.156*  (0.080) | -0.198**  (0.080) |
| Mothers’ ethnicity |  | 0.070  (0.082) | 0.072  (0.083) | 0.072  (0.082) | 0.150*  (0.080) |
| Mothers’ secondary education |  | -0.188* | -0.204*  (0.108) | -0.202*  (0.108) | -0.336***  (0.107) |
| Mothers’ third level education |  | -0.183*  (0.111) | -.0186*  (0.111) | -0.190*  (0.111) | -0.487***  (0.110) |
| Mothers’ have a disability |  | 0.325***  (0.061) | 0.325***  (0.061) | 0.323***  (0.061) | 0.326***  (0.059) |
| Fathers’ ethnicity |  | 0.142*  (0.080) | 0.131  (0.081) | 0.135*  (0.080) | 0.229***  (0.077) |
| Fathers’ secondary education |  | -0.147  (0.091) | -0.154*  (0.090) | -0.153*  (0.090) | -0.242***  (0.087) |
| Fathers’ third level education |  | -0.270***  (0.100) | -0.253**  (0.099) | -0.263***  (0.100) | -0.488***  (0.095) |
| Fathers’ have a disability |  | 0.170***  (0.066) | 0.179***  (0.066) | 0.177***  (0.066) | 0.235***  (0.064) |
| Other household member with chronic illness |  | 0.278**  (0.130) | 0.275**  (0.131) | 0.274**  (0.131) | 0.280**  (0.129) |
| Household living in rural area |  | -0.029  (0.041) | -0.039  (0.041) | -0.037  (0.041) | 0.049  (0.040) |
| Household live in rented accommodation |  | 0.301***  (0.066) | 0.311***  (0.065) | .3111713***  (.0660301) | .5194851***  (0.062) |
| Household number |  | 0.157***  (0.033) | 0.167***  (0.033) | 0.162***  (0.033) | 0.118***  (0.032) |
| Linktest |  | -.1137432*** | .0441095^NS^ | .0922396** | .0617906^NS^ |
| AIC |  | 5495.039 | 5465.914 | 5481.414 | 5779.535 |
| BIC |  | -640.442 | -662.804 | -654.068 | -342.418 |
| Pseudo R^2^ |  | 0.1314 | 0.1363 | 0.1336 | 0.0867 |
| Log likelihood |  | -2725.520 | -2709.957 | -2718.707 | -2865.768 |
| N |  | 6,400 | 6,400 | 6,400 | 6,400 |

***denotes significant at 1%, ** denotes significant at 5%, * denotes significant at 10%.

Results are based on probit model coefficients. Clustered standard error results are presented in parenthesis.

Table S12: OLS Estimates Using Weekly Household Income (Weighted)

| **Variables** | **Model 1 OLS**  **(I)** | **Model 2**  **OLS**  **(III)** |
| --- | --- | --- |
| **Children’s Health Status** |  |  |
| Children have no chronic illness/disability |  |  |
| Children have a chronic illness/disability | -86.918***  (27.797) |  |
| Children have a severe limitation in daily activities |  | -306.473***  (111.699) |
| Children have some limitation in daily activities |  | -106.876**  (48.586) |
| Children have no limitation in daily activities as a result of their condition |  | -61.1005*  (31.802) |
| **Mothers’ Characteristics** |  |  |
| ***Mothers’ Age*** | -299.372  (213.398) | -295.832  (213.741) |
| Mothers’ age squared | 8.235  (5.559) | 8.146  (5.567) |
| Mothers’ age cubed | -0.071  (0.048) | -0.070  (0.048) |
| ***Mothers’ ethnicity – Irish*** |  |  |
| Mothers’ ethnicity non-Irish | 52.656  (65.078) | 52.920  (65.120) |
| ***Mothers’ highest educational attainment*** |  |  |
| Mothers’ have a primary education or none |  |  |
| Mothers’ have a secondary level education | 81.98444*  (48.177) | 81.766*  (48.312) |
| Mothers’ have a third level education or higher | 295.901***  (51.276) | 294.845***  (51.369) |
| ***Mothers’ have no chronic illness or disability*** |  |  |
| Mothers’ have a chronic illness or disability | -35.320  (26.640) | -37.907  (26.807) |
| ***Mothers’ are not depressed*** |  |  |
| Mothers’ are depressed | 0.652  (21.652) | 0.666  (21.604) |
| ***Mothers’ not in paid work*** |  |  |
| Mothers’ in part-time paid work | -17.610  (51.642) | -19.571  (51.738) |
| Mothers’ in full-time paid work | 58.506**  (26.978) | 57.913**  (27.016) |
| **Fathers’ Characteristics** |  |  |
| ***Fathers’ Age*** | 6.365  (15.529) | 6.488  (15.536) |
| Fathers’ age squared | -0.082  (0.173) | -0.083  (0.173) |
| ***Fathers’ ethnicity – Irish*** |  |  |
| Fathers’ ethnicity non-Irish | -161.777***  (42.545) | -162.871***  (42.616) |
| ***Fathers’ highest educational attainment*** |  |  |
| Fathers’ have a primary education or none |  |  |
| Fathers’ have a secondary level education | 83.37384***  (29.5405) | 84.328***  (29.491) |
| Fathers’ have a third level education or higher | 320.064***  (37.743) | 321.359***  (37.707) |
| ***Fathers’ have no chronic illness or disability*** |  |  |
| Fathers’ have a chronic illness or disability | -23.365  (42.404) | -22.627  (42.362) |
| ***Fathers’ not in paid work*** |  |  |
| Fathers’ in part-time paid work | 85.0451  (99.654) | 86.116  (99.710) |
| Fathers’ in full-time paid work | 360.515***  (36.436) | 361.184***  (36.518) |
| **Household level characteristics** |  |  |
| Divorced/Separated/Single |  |  |
| Cohabitating with spouse/partner |  | 4.859  (32.881) |
| **Number of children** |  |  |
| 2 | -3.226  (41.463) | -1.019  (41.261) |
| 3 | -5.356  (42.5445) | -3.474  (42.343) |
| 4 | 70.785  (46.689) | 72.491  (46.415) |
| 5 | 81.418  (59.516) | 85.435  (59.385) |
| **Household members who do not have a chronic illness/disability** |  |  |
| Household members do have a chronic illness/disability | -92.360  (60.028) | -92.288  (60.042) |
| **Household living in urban area** |  |  |
| Household living in a rural area | -126.909***  (26.272) | -126.949***  (26.213) |
| **Household have no regular access to public transport** |  |  |
| Household have regular access to public transport | 35.716  (24.715) | 35.858  (24.711) |
| **Household living in non-rented accommodation** |  |  |
| Household lives in rented accommodation | -211.138***  (30.644) | -210.669***  (30.674) |
| **R-Squared** | 0.1782 | 0.1786 |
| **F-Statistic** | 25.67 | 23.93 |
| **N** | 3,759 | 3,759 |

***denotes significant at 1%, ** denotes significant at 5%, * denotes significant at 10%.

Sampling weights were applied due to the presence of heteroscedasticity. Clustered standard error results are presented in parenthesis.

Table S13: Binary Probit Model using Standard of Living Indicator – Does your Household Experience Difficulty Making Ends Meet (Weighted)

| **Coefficients** | **Model 1** | **Model 2** |
| --- | --- | --- |
| Children with a disability | 0.084  (0.113) |  |
| Children have a severe limitation in daily activities |  | 0.683  (0.513) |
| Children have some limitation in daily activities |  | 0.293  (0.180) |
| Children have no limitation in daily activities as a results of their condition |  | -0.138  (0.150) |
| Weekly household income residual | -0.001***  (0.0001) | -0.001***  (0.0001) |
| Weekly income squared residual | -8.25e-08  (1.85e-07) | -9.28e-08  (1.85e-07) |
| **Number of children** |  |  |
| **2** | 0.122  (0.153) | 0.104  (0.155) |
| **3** | 0.134  (0.169) | 0.115  (0.170) |
| **4** | 0.043  (0.202) | 0.017  (0.204) |
| **5** | 0.043  (0.2667) | 0.001  (0.269) |
| **Mothers’ ethnicity** | 0.040  (0.141) | 0.033  (0.142) |
| **Mothers’ highest educational attainment** |  |  |
| Mothers’ have a secondary level education | -0.616***  (0.176) | -0.610***  (0.176) |
| Mothers’ have a third level education or higher | -0.832***  (0.185) | -0.816***  (0.185) |
| **Mothers’ have a chronic illness/ disability** | 0.258**  (0.103) | 0.273***  (0.103) |
| **Fathers’ ethnicity** | 0.255*  (0.134) | 00.265**  (0.134) |
| **Fathers’ highest educational attainment** |  |  |
| Fathers’ have a secondary level education | -0.484***  (0.163) | -0.488*** (0.163) |
| Fathers’ have a third level education or higher | -0.773***  (0.174) | -0.778***  (0.174) |
| Fathers’ have a chronic illness/ disability | 0.268**  (0.123) | 0.267**  (0.124) |
| **Martial status** | -0.466***  (0.162) | -0.461***  (0.162) |
| **Household members who have a chronic illness/disability** | 0.388  (0.261) | 0.381  (0.261) |
| **Household number** | 0.211***  (0.052) | 0.215***  (0.052) |
| Household living in rural area | 0.104  (0.068) | 0.105  (0.068) |
| Household live in rented accommodation | 0.518***  (0.115) | 0.516***  (0.114) |
| AIC | 2713.749 | 2710.501 |
| BIC | -271.963 | -262.747 |
| Pseudo R^2^ | 0.1405 | 0.1428 |
| N | 3,759 | 3,759 |

***denotes significant at 1%, ** denotes significant at 5%, * denotes significant at 10%.

in addition to clustered standard errors

Table S14: Binary Probit Model using Standard of Living Indicator – Does your Household Experience Difficulty Making Ends Meet (Non-Weighted Using Two-Stage Residual Inclusion Estimates)

| **Coefficients** | **Model 1** | **Model 2** |
| --- | --- | --- |
| Children with a disability | -0.020  (0.108) |  |
| Children have a severe limitation in daily activities |  | 0.438  (0.475) |
| Children have some limitation in daily activities |  | 0.076  (0.125) |
| Children have no limitation in daily activities as a results of their condition |  | -0.106  (0.129) |
| Weekly household income | -0.002***  (0.0004) | -0.002***  (0.0004) |
| Weekly income squared | 1.31e-07  (1.53e-07) | 1.29e-07  (1.58e-07) |
| Weekly household income residual | 0 .0006*  (0.0003) | 0.0006**  (0.0003) |
| Weekly income squared residual | -8.47e-08  (1.95e-07) | -8.54e-08  (2.10e-07) |
| **Number of children** |  |  |
| **2** | 0.092  (0.173) | 0.086  (0.156) |
| **3** | 0.019  (0.189) | 0.013  (0.176) |
| **4** | 0.032  (0.217) | 0.022  (0.186) |
| **5** | -0.044  (0.292) | -0.061  (0.253) |
| **Mothers’ ethnicity** | 0.053  (0.114) | 0.053  (0.118) |
| **Mothers’ highest educational attainment** |  |  |
| Mothers’ have a secondary level education | -0.403**  (0.172) | -0.403**  (0.169) |
| Mothers’ have a third level education or higher | -0.320*  (0.182) | -0.316  (0.203) |
| **Mothers’ have a chronic illness/ disability** | 0.222**  (0.093) | 0.228**  (0.101) |
| **Fathers’ ethnicity** | -0.069  (0.115) | -0.065  (0.127) |
| **Fathers’ highest educational attainment** |  |  |
| Fathers’ have a secondary level education | -0.033  (0.173) | -0.034  (0.149) |
| Fathers’ have a third level education or higher | 0.153  (0.223) | 0.148  (0.200) |
| Fathers’ have a chronic illness/ disability | 0.107  (0.119) | 0.105  (0.092) |
| **Martial status** | -0.221*  (0.119) | -0.220*  (0.119) |
| **Household members who have a chronic illness/disability** | 0.167  (0.198) | 0.162  (0.274) |
| **Household number** | 0.245***  (0.060) | 0.247***  (0.050) |
| Household living in rural area | -0.139**  (0.070) | -0.138**  (0.062) |
| Household live in rented accommodation | 0.102  (0.142) | 0.102  (0.126) |
| AIC | 2730.050 | 2732.134 |
| BIC | -292.409 | -277.861 |
| Pseudo R^2^ | 0.1500 | 0.1506 |
| N | 3,759 | 3,759 |

***denotes significant at 1%, ** denotes significant at 5%, * denotes significant at 10%.

in addition to bootstrapped standard errors

1. Variables for Mothers’ age squared and age cubed are also included [↑](#footnote-ref-1)
2. A variable for Fathers’ age squared is also included [↑](#footnote-ref-2)
